# Epidemiology of *Cysticercus tenuicollis* in Sheep and Goats in the Tropics: A Systematic Review

**DOI:** 10.1155/vmi/7881494

**Published:** 2024-12-14

**Authors:** Prudentia Yensi Lawan, Aziwo Tatanja Niba, Julius Awah-Ndukum

**Affiliations:** Department of Animal Production Technology, College of Technology, The University of Bamenda, P.O. Box 39, Bambili, Bamenda, Cameroon

**Keywords:** *Cysticercus tenuicollis*, epidemiology, goats, sheep, systematic review, tropics

## Abstract

Sheep and goat influence the socioeconomic sustainability of rural communities in tropical countries, but parasitic diseases and *Cysticercus tenuicollis* in particular are responsible for their low productivity. The occurrence of *Cysticercus tenuicollis* in most affected regions of the world has been associated to the presence of stray dogs in the grazing area, which play vital roles in the life cycle of the parasite and poor disposal of contaminated and seized offal, organs, and carcasses. The aim of this paper is to systematically review the epidemiology of *Cysticercus tenuicollis* infection in sheep and goats in the tropics between 2010 and 2022 through the analysis of published qualitative and quantitative data on prevalence, risk factors, organ distribution, and interventions. The review showed that goats are more susceptible to *Cysticercus tenuicollis* than sheep in the tropics, and the prevalence rates based on meat inspection range from 0.45% to 56.8% in sheep and 4.83% to 72.38% in goats. More adult (2.9% to 83.17%) and female (0.96% to 71.42%) were infected than young (2.7% to 61.79%) and male (2.3% to 72.53%) animals. Higher infection rates were reported among animals in highland (7.99% to 73.61%) compared to animals in lowlands (4.70% to 69.69%). Overall, *Cysticercus tenuicollis* were observed on the abdominal visceral organs but predominantly on the omentum and liver, and affected animals were significantly higher during wet seasons. The review highlights the implementation of the One Health approach to improve understanding of the epidemiology, roles of different hosts, and environmental health in relation to the prevention and control of *Cysticercus tenuicollis* in the tropics.

## 1. Introduction

Livestock production is an integral part of the agricultural production systems and development of the economy, employment, food, and nutritional security as well as cultural events such as births, deaths, and marriages in communities in the tropics [1, 2]. However, the full exploitation of these resources has been hindered by many factors such as drought, poor genetic potential, backward animal husbandry practice, and the prevalence of diseases [3]. Parasitic diseases pose serious concerns at the human–animal interface in many tropical and developing countries [4, 5] and cause significant morbidity and mortality in humans and animals [6–8].

Cysticercosis is a neglected parasitic infection [9] and an underestimated zoonosis [10, 11] at the human–animal interface. Though the clinical presentation of *Taenia hydatigena*, cysticercosis is highly unspecific and could be symptomless, and it can be diagnosed with a specificity of 100% by postmortem examination [12]. Occasionally, clinical signs such as thriftiness, diarrhea, respiratory signs, and convulsions due by *Cysticercus tenuicollis* have been described [11, 13]. However, the early-stage *Taenia hydatigena* metacestodes are small and may be missed during postmortem examinations [12]. The adult dog tapeworm, taeniid cestodes *Taenia hydatigena*, are found in the small intestines of dogs, cats, and wild canids [5] while the intermediate hosts for the metacestodes *Cysticercus tenuicollis* inhabit in domestic (sheep, goats, cattle, pigs, and horses) and wild ruminants hosts (antelopes and deer) [11, 14–16]. However, the prevalence of the *Cysticercus tenuicollis* infection vary according to geographical areas with higher incidences observed in countries with low levels of sanitary control and uncontrolled movements of wild carnivores population [5, 17]. Geographical, environmental, meteorological, and managerial factors are crucial to the life cycle of the helminths and significantly influence prevalence rates of infection of the metacestodes [18, 19]. Other factors such as age, sex, breed, body condition score, origin, and physiological state may influence the innate susceptibility or resistance of hosts to the infection [20]. Cysticercosis is a focal disease, affecting the poor communities especially where hygiene practices are deficient. Stray dogs are important reservoirs for the transmission of zoonotic helminths including cysticercosis, and large populations of stray dogs increase the risk of spreading the disease [19, 21], at the human–animal interface.

There are great contacts between rural communities with dogs and domestic animals which are the main reservoir for metacestodes [22]. Infection with cysts of *Cysticercus tenuicollis* causes high degree of morbidity and mortality in livestock [22, 23]. Cysticercosis have public health and veterinary significances and cause enormous socioeconomic losses to subsistence farming communities and gross financial losses due to condemnation of infected offal and meat during slaughter inspections [5, 15, 16, 22, 24, 25] in developing countries such as Africa, Asia, and Latin America.

The standard diagnostic technique of *Cysticercus tenuicollis* in livestock is by detection of the cyst at postmortem examination [19, 26] and use of serological techniques with cautious analysis of biochemical and hematological tests on live animals [12, 26]. The predominant body sites of larval stage of *Cysticercus tenuicollis* are the surfaces of abdominal visceral structures such as the liver, spleen, omentum, kidney, heart, and mesentery [6, 12, 17, 27–29]. However, *Cysticercus tenuicollis* have also been detected on other body parts including lungs, pleura, pericardium, kidneys, brain, and reproductive system such as ovaries, uterus, uterine tubes, cervix, and vagina [19, 30].

Though the prevalence of *Cysticercus tenuicollis* infection in small ruminants is high and infection rates of over 40% among slaughtered sheep and goats have been reported in many countries in Africa [3, 5, 12, 17, 18, 23, 26, 31], there is scanty information on the epidemiology and distribution of the disease in the African continent [16]. Tolossa et al. [26] have associated the effect of *Cysticercus tenuicollis* infection on the hosts to the degree of the parasitism, organs involved, and existence of other concurrent infections. However, the impact of *Cysticercus tenuicollis* infection on the health of humans and animals and livestock production systems in the continent is not known. Cysticercosis due to *Cysticercus tenuicollis* is a neglected disease worldwide and can pose serious public health concerns particularly in the tropical zones [12]. Therefore, the aim of this report was to systematically review of the prevalence estimate, diagnostic methods, associated risk factors, organ distribution, and control measures as well as associated financial loss of *Cysticercus tenuicollis* infection in sheep and goats.

## 2. Methods

### 2.1. Study Design and Search Strategy

Following the conception of the systematic review and identification of a title, a protocol was prepared based on guidelines of the Preferred Reporting Items for Systematic reviews and Meta-Analyses (PRISMA) [32]. A systematic thorough search was conducted of peer-reviewed literature reporting on the prevalence, associated risk factors, organ distribution, financial losses and control measures, and strategies of *Cysticercus tenuicollis* infection of sheep and goats in the tropics. The search was conducted through Web of Science, Scopus, PubMed, Academic Search Complete, Google Scholar, and African Journal Online (AJOL). These databases were explored using combinations of the following keywords and phrases: (“Prevalence” OR “Epidemiology”) AND (“Risk factors” OR “Control strategies” OR “Challenges for control” OR “Control”) AND (“Sheep” OR “Ovine” OR “Goats” OR “Caprine” OR “Wildlife” OR “Animals”) AND (“Caprine Cysticercosis” OR “Ovine Cysticercosis” OR “Cysticercosis” OR “*Cysticercus tenuicollis* infection” OR “Metacestodes” OR “Taenid cestodes” OR “*Taenia hydatigena*”) AND (“Diagnostic methods” OR “Diagnosis” OR “Post mortem examination” OR “Cyst detection” OR “meat inspection” OR “Abattoir meat inspection” OR “Slaughterhouse survey”) AND (“Organ distribution” OR “Body organ” OR “Organ affinity”) AND (“Economic loss” OR “Financial loss” OR “Social impact” OR “Impact” OR “Consequence” OR “Public health” OR “Zoonosis”) AND (“Africa” OR “Asia” OR “Tropics” OR “Developing countries”). Furthermore, a search was conducted using the key terms in Google Chrome while authors of relevant references cited on papers chosen for this review, and researchers who have published on *Cysticercus tenuicollis* infection in sheep and goats in the tropics were contacted. However, the time restriction as regards the publication date during these searches was between 2010 and 2022. The results of the search were restricted on the type of journal, period, and/or duration of study. Mendeley Desktop online software was used to key in the citations for easy access. Finally, reference lists of reviews were screened for inclusiveness and additional relevant records added to the database. The search was conducted during the period of 4th to 17th July 2022.

### 2.2. Selection Criteria

This review included all studies on *Cysticercus tenuicollis* in sheep and goats in the tropics. The review included studies on prevalence estimate, diagnostic methods, associated risk factors, organ distribution, and control measures as well as associated financial loss of *Cysticercus tenuicollis* infection in sheep and goats in the tropical countries. Thus, during the compilation of the search results from the different literature, focus was made on keywords and phrases used and the period of the study in each paper. Original articles on cross-sectional studies from the tropics on the basis of their title and abstracts relevant to the research objectives were included in the study. Duplicated records were removed. Titles and abstract were screened for relevance applying the following inclusive criteria: (i) studies conducted during the defined period between 2010 and 2022; (ii) studies conducted in tropical areas, (iii) studies concerning *Cysticercus tenuicollis* in sheep and goats; (iv) studies reporting results within the scope of prevalence estimate, diagnostic methods, associated risk factors, organ distribution, and control measures of *Cysticercus tenuicollis* infection in sheep and goats; (v) studies reported in English; and (vi) full text was available which was evaluated and reference checked to ensure that all the required information was available for entry. However, studies that did not contain the relevant data were excluded from the review. Two independent reviewers conducted the screening process, and a third reviewer decided on areas of disagreement between the two reviewers.

### 2.3. Data Extraction

Papers were read and screened to ensure the required data were available for extraction. Data were extracted from individual study using a form, and database was developed for the purposes of this review using Microsoft Excel 2010. The data extraction was independently done by two coauthors (LPY and ATN). Discrepancies between the coauthors were resolved by discussion, and in cases where that was not possible, a third reviewer (JAN) resolved them. Articles that met the inclusion criteria and reported data on the prevalence, diagnostic methods, associated risk factors, organ distribution, and control measures of *Cysticercus tenuicollis* infection in sheep and goats were included in the systematic review. Information extracted included article information (first author, year of publication, duration of study, and location) and study design (samples size, cross-sectional design, or longitudinal study). The data were extracted from text, tables, and graphs and, in some cases, in the narratives of the text.

### 2.4. Data Analysis

The quality of the selected papers for the review was evaluated using previously described critical appraisal checklists [32–34] for prevalence and case report studies. Briefly, the appraisal checklist included the following: (i) appropriate sampling frame; (ii) proper sampling technique; (iii) adequate sample size; (iv) adequate description of study subjects and setting; (v) sufficient data analysis; (vi) use of valid detection techniques for the identified conditions; (vii) adequate training of those involved in the detection of the identified conditions; and (viii) use of appropriate statistical analysis (for prevalence). Based on the described appraisal checklist, Yes (Y), No (N), unclear (U), or not applicable (NA) were assigned to answer each question on the checklists. Articles with ≤ 60% score were considered low-quality studies. Two reviewers conducted the quality assessment, and the third reviewer resolved discrepancies.

In papers wherein only the number of animals examined were available as well as the prevalence, the number of animals affected was calculated [35] and inserted. In other cases, the prevalence was calculated when the number of animals examined (denominator) and the number of animals infected (numerator) were indicated [11, 35]. Synthesis of the findings was also done to group the outcomes of the different authors to avoid repetition. Where information was lacking, the space was filled with NA, meaning not available.

Following quality assessment of selected articles using the critical appraisal checklist, the obtained data on prevalence estimates, diagnostic methods, risk factors, organ distribution, and control measures of *Cysticercus tenuicollis* infection in sheep and goats and the associated financial losses were pooled into Microsoft Excel 2010 for systematic descriptive analysis. Prevalence was estimated as previously described [11, 35].

## 3. Results and Discussion

### 3.1. General Results

**Figure 1:**
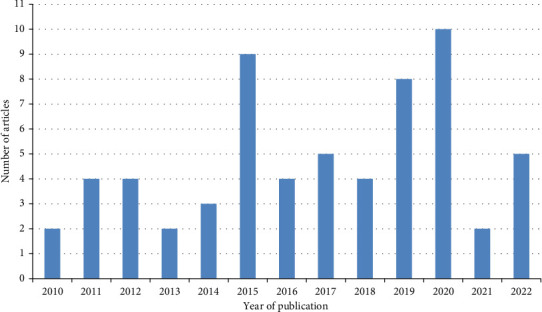
Number of *Cysticercus tenuicollis* infection in sheep and goats publications per year in the tropics (2010–2022) that were included in the study.

**Figure 2:**
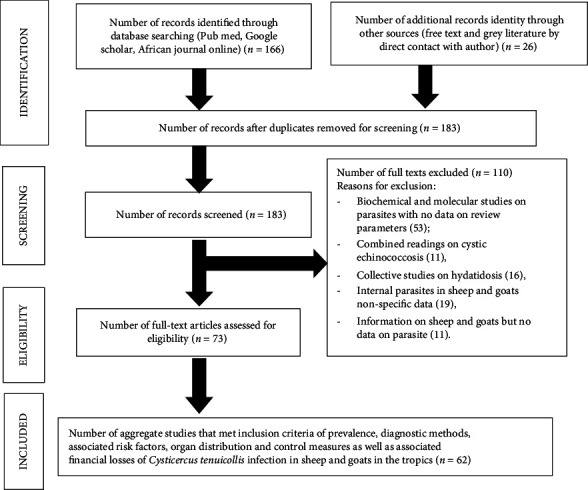
PRISMA diagram for a systemic review of *Cysticercus tenuicollis* in sheep and goats in the tropics researched from 2010–2022. *Source:* Adopted and modified from [36, 37].

A total of 166 scientific articles were retrieved following initial search of online databases. Additional 26 articles were obtained by free online search (13), contacting authors who have studies on *Cysticercus tenuicollis* infection in sheep and goats in the tropics (5), searching reference lists of included studies (8) and local database (0). A total of nine duplicates were removed, and 183 articles screened for eligibility on the basis title and abstracts. Overall, 110 records were excluded for nonrelevance to the research objectives, and 73 full-text articles were assessed for eligibility with 62 articles meeting the inclusion criteria (score of > 60% on critical checklist appraisal assessment) for the study (Figures 1 and 2).

The highest number of publications were 2020 (10) followed by 2015 (9), 2019 (8), 2017 (5), and 2022 (5) and the lowest from 2010 (2), 2013 (2), and 2021 (2) compared to other years (Figure 1). The tropical regions where the studies were done included Africa (43), Asia (08), and Latin America (01) with the highest number of publications retrieved from Ethiopia (15) followed by Iraq (06) and Egypt (05). Of the 62 articles used in the review (Figures 1 and 2), 42 were rated as good quality (low risk of bias) and 20 were of moderate quality (medium risk of bias) and assessed prevalence estimates (40), diagnostic methods (37), associated risk factors (35), organ distribution (33), and control measures (34) as well as associated financial losses (5) of *Cysticercus tenuicollis* infection in sheep and goats in the tropics (Tables 1, 2, 3, 4, 5, 6, 7, and 8).

### 3.2. Epidemiology of *Cysticercus tenuicollis* Infection in Sheep and Goats

The reviewed studies adopted various study designs, diagnostic tests, and target groups to estimate the prevalence, associated risk factors, organ distribution, and control measures of *Cysticercus tenuicollis* infection of sheep and goats in different tropical regions. This review presents the study results in situ without comparing levels of infections between studies, sites, or periods. Though cysticercosis due to *Cysticercus tenuicollis* in sheep and goats is usually asymptomatic [66], meat inspection of slaughtered live animals and postmortem of death animals for the detection of metacestodes are the main diagnostic procedures used [19, 26, 27, 39]. In the present study, *Cysticercus tenuicollis* infection in sheep and goats is widespread in 25 tropical countries in Africa (46), Asia (8), and South America (1), and its prevalence varies in magnitude between countries and hosts. Overall, the prevalence of *Cysticercus tenuicollis* in sheep and goats in the tropics ranged from 4.83% to 72.38%, with higher values observed in Africa (particularly in Ethiopia) compared to Asia [67]. The present review included 9 studies in Ethiopia showing highest prevalence for the diseases in goats (72.38%) and in sheep (46.69%) in the country. Also, high prevalence (over 45.7%) of *Cysticercus tenuicollis* in goats was reported in Tanzania [12, 18]. The presence of the disease cannot be excluded in other countries given that there is widespread rearing of small ruminants, uncontrolled livestock movements across borders of neighboring countries, and lack of diagnosis and reporting does not exclude the presence of the disease in many tropical regions.

However, the differences in prevalence in the review (Table 1) was associated to the variation in climate and season [68], geographical characteristics of locations and husbandry management system of the animals [3], level of input in the management system and size of the small ruminant livestock population [18, 26], and frequency and extend of the epidemiological studies [42].

### 3.3. Associated Risk Factors *Cysticercus tenuicollis* in Sheep and Goats in the Tropics

Few studies focused on the effect of sex, age, season, and geographical epidemiology of *Cysticercus tenuicollis* in sheep and goats.

#### 3.3.1. Age

Table 2 presents the distribution of *Cysticercus tenuicollis* of sheep and goats in some tropical countries according to age. High prevalence was reported in Ethiopia for the disease in adult goats (83.17%) [42] and sheep (80.4%) [26] suggesting that duration (age of animal) and nature of exposure are important risk factors. The adult animals were exposed to potential sources of the infection for long periods and at varying levels depending on the level and nature of environmental contaminations than younger animals. However, goats usually show more infections with internal parasites than sheep [58]. Traditional husbandry practices, mixing of livestock in same microenvironments (such as focal points for veterinary service deliveries, livestock markets, watering spots, and communal grazing fields among others) and extensive grazing patterns, are widely practiced in tropical areas [53, 59]. Also, young animals (postweaning), lactating animals, and animals in late gestation or at time of parturition have increased susceptibility to diseases including parasitism due to underdeveloped or dropped immune status [69, 70].

#### 3.3.2. Sex

Overall, *Cysticercus tenuicollis* prevalence range of 2.3% to 72.53% in the male sheep and goat and 0.96% to 71.42% in female sheep and goat was recorded (Table 3). The range was 0.96% to 61.17% in female sheep, 5.97% to 71.42% in female goats, 0.96% to 45.43% in male sheep, and 5.97% to 72.53% in male goats (Table 4). The highest prevalence of the disease was recorded in the male goat (72.53%) and female goats (71.42%) by [42] in Ethiopia. The higher prevalence of the disease in female than in male animals was associated to physiological stresses in females such as pregnancy and lactation which dropped their immune status as well as longer lifespans of female in farms than in the male animals [59].

#### 3.3.3. Altitude of Geographical Location

The review showed that the prevalence ranged from 4.70% to 69.69% for sheep and goats in lowland areas compared to 7.99% to 73.61% for sheep and goats in highland areas (Table 5). More specifically, the prevalence range from 20.11% to 73.61% for goats in highlands is compared to 4.7% to 69.69% for goats in lowlands against a prevalence range from 7.99% to 60.07% for sheep in highlands and 6.82% to 53.47% for sheep in the lowland areas. However, geographical location and altitude did not seem to influence the prevalence rate of *Cysticercus tenuicollis* in sheep and goats. The observed variation of the prevalence in the lowland and highland areas was associated to dissemination of segments of *Taenia hydatigena* infection by the final host animal (especially infected stray dogs) in small ruminant farms [70]. Also, traditional and nomadic livestock farming systems, geographic characteristics of the environments, and meteorological and managerial factors were reported to significantly influence the life cycle of *Taenia hydatigena/Cysticercus tenuicollis* [5, 17, 18].

#### 3.3.4. Season

The period of the year and season influenced the prevalence of *Cysticercus tenuicollis* infection in sheep and goats in the tropics (Table 5). Overall, the highest prevalence rates were recorded during the early wet or rainy season, followed by the late wet or rainy season. This was closely followed by the peak of the rainy season and the peak of the dry season. The variation of the prevalence with season could be due to seasonal migration of animals including *Taenia hydatigena/Cysticercus tenuicollis* infected animals and the disease transmission between contaminated and clean communities.

However, male dogs roamed more than female dogs during the early periods (i.e., spring-March, April, and May) and the late periods (September, October, and November) of wet seasons [56]. These periods correspond to breeding periods and physiological changes in bitches such as estrous (heat) and provide favorable conditions for the dissemination of eggs/segments of parasites.

### 3.4. Distribution of *Cysticercus tenuicollis* on Visceral Organs of Infected Sheep and Goats in the Tropics

The review showed that *Cysticercus tenuicollis* were typically found on abdominal visceral organs such as the omentum, liver, mesentery, and peritoneum [6, 19, 27–29, 41]. The predominant predilection site was the omentum due to its vast size and the fact that it covers most abdominal visceral organs, followed by the liver due to its involvement in the life cycle (Table 6). Condemnation of the affected organs during meat inspection negatively impacted productivity due to the disease and caused significant direct financial and economic losses [3]. There were negative socioeconomic impacts due to *Cysticercus tenuicollis* and consequence on poverty alleviation due to low economic output to small ruminant farmers, butchers, and concerned communities whose livelihood depend on sheep and goat production [46]. The lack of integrated surveillance systems, underreporting, and inefficiencies and the lack of control measure of *Cysticercus tenuicollis* have also been highlighted [71]. This probably explains why parasitic diseases are responsible for great losses in the meat industries in the tropics than other infectious and metabolic diseases [43].

### 3.5. Challenges to Control of *Cysticercus tenuicollis* Infection in Sheep and Goats

The review observed that the widespread presence of intermediate host and final hosts which are vital for the life cycle of the parasite is the main reason for the variations in the prevalence and the persistence of *Taenia hydatigena/Cysticercus tenuicollis* infection in sheep and goats in the tropics (Table 7). Several studies reported that the presence of stray dogs which played vital roles in the life cycle of the parasite includes those of [5, 7, 11, 15, 23, 26, 36, 38, 40, 41]. Therefore, avoidance and control of clandestine/illegal slaughtering of animals without veterinary supervision, adequate disposal of abattoir, and other related contaminated materials and control of stray dogs significantly interrupted the life cycle of the parasite and reduced infection [38, 64]. Also, good husbandry management and environmental hygiene practices, adequate veterinary services, good nutrition, and feeding and adequate immune status were cited as control measures of the disease [3, 41]. Therefore, the key preventive approaches in reducing the prevalence of *Cysticercus tenuicollis* in sheep and goats focused on the regulation of stray dogs and proper disposal of contaminated materials (Table 8).

Overall, the articles included in this study presented relevant information for the systematic review including study identifier (authors names, year of publication, and study identification) and prevalence estimates in various subgroups (age, sex, geographical location, season, and organs distribution), diagnostic methods, associated risk factors, control measures and challenges, and associated financial loss of *Cysticercus tenuicollis* infection in sheep and goats. However, most of the articles used in this review lacked a measure of variability such as the absence of confidence intervals for better understanding the precision of prevalence estimates.

## 4. Conclusion

*Cysticercus tenuicollis* infection in sheep and goats is widespread but neglected in many African countries including Cameroon. The predominant diagnostic method was postmortem inspection of slaughtered sheep and goats. Condemnation of the affected organs during meat inspection impacted negatively on productivity and caused significant direct financial and economic losses. Preventive approaches of *Cysticercus tenuicollis* infection in sheep and goats should focus on the regulation of stray dogs and proper disposal of contaminated materials. Also, public health education, continuous disease surveillance, and instruction of stakeholders on husbandry management are vital to reduce the burden of *Cysticercus tenuicollis* infection in sheep and goats in endemic regions [72].

## Figures and Tables

**Table 1:** Diagnostic methods and prevalence of *Cysticercus tenuicollis* infection in sheep and goats in the tropics.

Country	Area of study	Diagnostic method	Sheep		Goat		Reference
			Number	Prevalence (%)	Number	Prevalence (%)	
Ethiopia	Central Ethiopia	Postmortem examination	252	40.0	358	46.6	[17]
Iraq	Basrah	Postmortem examination	180	40.55	90	26.25	[38]
Nigeria	Sokoto	Postmortem inspection	261	13.03	NA	NA	[39]
Ethiopia	Abattoirs of Ethiopia	Postmortem examination	576	56.8	576	63.9	[23]
Iran	Fars, Southern Iran	Postmortem examination	184	17.52	523	55.05	[26]
Ethiopia	Addis Ababa	Postmortem inspection	576	7.81	576	15.8	[40]
Ethiopia	DiriDawa	Postmortem inspection	425	22.8	420	26.4	[41]
Ethiopia	Dessie	Postmortem examination	510	46.69	420	72.38	[42]
Iran	Kermanshah	Meat inspection	5768	5.58	647	8.04	[7]
Ethiopia	Bishoftu, Elfora Abattoir	Meat inspection	162	45	280	53.9	[43]
Tanzania	Mbeya, Tanzania	Postmortem inspection	27	45.7	392	51.9	[12]
Iraq	Karbala	Postmortem inspection	240	32.5	240	35.41	[44]
Uganda	Soroti	Postmortem inspection	160	42.5	294	33.3	[45]
India	Northern India	Postmortem inspection	760	2.23	2439	4.83	[27]
Ethiopia	Helimex Abattoir	Postmortem inspection	384	11.46	384	12.76	[46]
Ethiopia	Addis Ababa Abattoir	Postmortem inspection	600	37.02	300	44.0	[3]
Ethiopia	Debrezeit town	Postmortem inspection	576	56.8	576	63.9	[47]
Egypt	Aswan	Postmortem inspection	669	13.3	484	24.2	[28]
Tanzania	Malambo slaughter slab	Postmortem examination	38	42.2	55	61.1	[18]
Tanzania	Mbeya and Rukwa regions	Postmortem examination	27	51.9	392	45.7	[12]
Brazil	Slaughterhouses in municipalities of Patos and Esperança, Paraíba	Postmortem inspection	195	17.4	195	39	[4]
Algeria	El Tarf abattoir	Postmortem examination, biochemical analysis	256	24.21	164	43.90	[31]
Saudi Arabia	Al Kakee's Slaughter Makkah	Postmortem examination	1099	0.45	477	23.4	[48]
Algeria	Algeria	Postmortem examination, biochemical and morphological characterizations	1973	7.8	1175	22.3	[15]
Ethiopia	Bishoftu Elfora	Postmortem inspection	232	35.4	268	54.2	[5]
Ethiopia	Jimma municipal abattoir	Postmortem inspection	275	16.72	109	12.84	[49]
Tunisia	Northeast	Postmortem examination	3692	2.8	78	8.9	[11]
Ethiopia	Central Oromia	Postmortem examination	90	48.9	310	30.6	[26]
Iraq	Al-Diwaniyah abattoirs	Postmortem examination	426	11.73	210	2.85	[50]
Iran	Darab Slaughterhouses	Postmortem inspection	646	8.4	2029	5.5	[29]
Egypt	Matrouh city abattoir	Postmortem inspection	1400	11	2816	21	[51]
Iraq	Kalar district	Postmortem inspection	2906	6.88	NA	NA	[52]
Ethiopia	Abyssinia exort abattoir, Bishoftu	Postmortem examination	64	3.65	320	16.9	[53]
Tunisia	Northeast	Postmortem examination	3692	2.8	78	8.9	[11]
Iraq	Sulaymaniyah province		19,897	22.6	NA	NA	[19]
Ghana	Tamale metropolis abattoir, North region	Postmortem inspection	256	22.66	538	22.34	[54]
Egypt	General Mersa Matrouh abattoir	Postmortem inspection	555	12	1431	22	[55]
Iraq	Al-Shuala abattoir, Northern Baghdad	Postmortem inspection	100	21	100	35	[56]
Algeria	El Harrach and Boufarik		461	4.31	241	2.25	[57]
Iraq	Basrah province slaughterhouse	Postmortem inspection	23	2.6	23	2.6	[58]
Bangladesh	Slaughterhouses at, Bramhapalli, Jubileeghat, Mesuabazar, Mymensinghsadar, Mymensingh	Postmortem inspection	NA	NA	1372	12.9	[59]
Saudi Arabia	Municipal abattoir of Makkah	Postmortem inspection	23,542	4.95	554	4.75	[60]
Cameroon	NA		NA	0	NA	0	NA

Abbreviation: NA, not available.

**Table 2:** Prevalence of *Cysticercus tenuicollis* according to the age of sheep and goats in the tropics.

Country	Area of study	Species	Age	No. examined	No infected	Prevalence (%)	Source
Ethiopia	Central Ethiopia	Ovine	Young	NA	NA	35.8	[17]
			Adult	NA	NA	47.4	
		Caprine	Young	NA	NA	41.4	
			Adult	NA	NA	51.8	

Ethiopia	Abattoirs in Ethiopia	Ovine	Young	288	150	52.1	[23]
			Adult	288	177	61.5	
		Caprine	Young	288	170	59.03	
			Adult	288	198	68.8	

Ethiopia	Addis Ababa	Ovine	Young	119	05	4.2	[44]
			Adult	457	40	8.75	
		Caprine	Young	157	13	8.28	
			Adult	419	78	18.62	

Ethiopia	Dire Dawa municipal abattoir	Ovine	Young	211	54	25.6	[41]
			Adult	214	43	20.1	
		Caprine	Young	39	9	23.1	
			Adult	381	102	26.8	

Ethiopia	Dessie municipal abattoir	Ovine	Young	234	87	37.18	[42]
			Adult	276	146	52.89	
		Caprine	Young	212	131	61.79	
			Adult	208	173	83.17	

Ethiopia	Bishoftu, Elfora abattoir	Ovine	Young	124	43	34.7	[5]
			Adult	138	75	54.3	
		Caprine	Young	123	57	46.3	
			Adult	157	94	59.9	

Ethiopia	Helimex Abattoir	Ovine	Young	192	21	10.94	[46]
			Adult	192	23	11.98	
		Caprine	Young	192	21	10.94	
			Adult	192	28	14.58	

Ethiopia	Addis Ababa abattoir	Ovine	Young	300	87	29	[3]
			Adult	300	136	45.3	
		Caprine	Young	NA	NA	NA	
			Adult	300	132	44	

Egypt	Aswan slaughterhouse	Ovine	Young	424	51	12.03	[28]
			Adult	245	38	15.5	
		Caprine	Young	448	108	24.1	
			Adult	36	9	25	

Tanzania	Ngorongoro, malambo slaughter slab	Ovine	< 1 year	14	5	35.7	[18]
			1–2 years	19	8	42.1	
			> 2 years	57	25	43.8	
		Caprine	< 1 year	22	11	50.0	
			1–2 years	20	15	75.0	
			> 2 years	48	29	60.4	

Ethiopia	Jimma municipal abattoir	Ovine	Young	133	26	19.5	[44]
			Adult	142	20	14.1	
		Caprine	Young	49	4	8.16	
			Adult	60	10	16.7	

Tunisia	Northeast Tunisia	Ovine	< 3 years	3133	86	2.7	[11]
			> 3 to < 6 years	35	5	14.3	
			> 6 years	524	15	2.9	
		Caprine	< 3 years	78	7	8.9	
			> 3 to < 6 years	NA	NA	NA	
			> 6 years	NA	NA	NA	

Iraq	Sulaymaniyah province	Ovine	Young	6533	1427	21.8	[19]
			Adult	13,364	3068	22.9	

Ethiopia	Central Oromia	Ovine	Young	90	NA	15.9	[26]
			Adult	NA	NA	80.4	
		Caprine	Young	310	NA	19.2	
			Adult	NA	NA	42.2	

Ethiopia	Bishoftu Elfora	Ovine	Young	NA	NA	41.0	[5]
			Adult	NA	NA	20.8	

Ghana	Tamale metropolis abattoir	Ovine	Young	NA	256	30.0	[57]
			Adult	NA	NA	23.0	
		Caprine	Young	NA	273	20.0	
			Adult	NA	NA	35.0	

Iraq	Al-Shuala abattoir, Northeast region, Baghdad city	Ovine	< 6 months	38	04	10.5	[59]
			< 6 to < 12 months	55	14	25.4	
			> 12 months	07	03	42.8	
		Caprine	< 6 months	42	14	33.3	
			< 6 to < 12 months	22	08	36.3	
			> 12 months	36	13	36.1	

Bangladesh	Slaughterhouses at brahmapalli, Jubilee Ghat, mesua bazar mymensingh sadar, mymensingh	Caprine	Adults (> 12 months)	430	89	9.3	[59]
			Young (< 12 months)	942	88	20.7	

Abbreviation: NA, not available.

**Table 3:** Prevalence of *Cysticercus tenuicollis* according to sex of sheep and goats in the tropics.

Country	Area of study	Specie	Sex	No. examined	No infected	Prevalence in sheep (%)	Source
Iraq	Basrah	Ovine	Male	95	21	22.10	[38]
			Female	85	58	61.17	
		Caprine	Male	51	11	21.56	
			Female	39	10	25.64	

Ethiopia	Dire Dawa municipal abattoir	Ovine	Male	211	54	25.6	[41]
			Female	214	43	20.1	
		Caprine	Male	228	58	25.4	
			Female	192	53	27.6	

Ethiopia	Addis Ababa	Ovine	Male	95	21	22.1	[40]
			Female	85	58	61.17	
		Caprine	Male	51	11	21.56	
			Female	39	10	25.64	

Ethiopia	Dessie municipal abattoir	Ovine	Male	416	189	45.43	[42]
			Female	94	44	46.81	
		Caprine	Male	364	264	72.53	
			Female	56	40	71.42	

Iran	Kermanshah	Ovine	Male	4077	205	5.03	[7]
			Female	1691	117	6.92	
		Caprine	Male	436	26	5.97	
			Female	211	26	12.32	

Ethiopia	Addis Ababa	Ovine	Male	300	107	35.7	[3]
			Female	300	116	38.7	
		Caprine	Male	NA	NA	NA	
			Female	150	132	44	

Egypt	Aswan slaughterhouse	Ovine	Male	441	24	5.4	[28]
			Female	228	65	28.5	
		Caprine	Male	424	89	20.9	
			Female	60	28	46.7	

Tanzania	Ngorongoro, malambo slaughter slab	Ovine	Male	60	24	40.0	[18]
			Female	30	14	46.7	
		Caprine	Male	41	8	19.5	
			Female	49	12	24.5	

Algeria	Tiaret abattoir	Ovine	Male	1973	136	6.8	[15]
			Female	1973	19	0.96	
		Caprine	Male	1175	155	13.1	
			Female	1175	108	9.19	

Ethiopia	Jimma municipal abattoir	Ovine	Male	166	27	16.26	[49]
			Female	109	19	17.43	
		Caprine	Male	63	5	7.9	
			Female	46	9	19.56	

Tunisia	Northeast Tunisia	Ovine	Male	1739	40	2.3	[11]
			Female	1953	66	3.4	
		Caprine	Male	46	3	6.5	
			Female	32	4	12.5	

Iraq	Sulaymaniyah province	Ovine	Male	16,697	3797	22.7	[31]
			Female	3200	698	21.8	

Ghana	Tamale metropolis abattoir, northern region	Ovine	Male	116	28	24.14	[57]
			Female	140	30	21.43	
		Caprine	Male	214	49	22.90	
			Female	59	12	20.34	

Iraq	Al-Shuala abattoir, northeast region, Baghdad city	Ovine	Male	84	18	21.42	[56]
			Female	16	03	18.75	
		Caprine	Male	100	35	35	
			Female	0	0	0	

Bangladesh	Slaughterhouses at brahmapalli, Jubilee Ghat, mesuabazar mymensinghsadar, mymensingh	Caprine	Male	811	71	8.8	[59]
			Female	561	106	18.9	

Abbreviation: NA, not available.

**Table 4:** Prevalence of *Cysticercus tenuicollis* according to altitude of location of sheep and goats in the tropics.

Country	Area of study	Origin	Animal study	No. examined	No infected	Prevalence (%)	Source
Ethiopia	Abattoirs in Ethiopia	Highlands	Sheep	288	173	60.07	[23]
		Lowlands	Sheep	288	154	53.47	
		Highlands	Goat	288	196	68.06	
		Lowlands	Goat	288	172	59.72	

Ethiopia	Addis Ababa	Highlands	Sheep	488	39	7.99	[44]
		Lowlands	Sheep	88	6	6.82	
		Highlands	Goat	179	36	20.11	
		Lowlands	Goat	397	55	13.85	

Ethiopia	Dessie municipal abattoir	Highlands	Sheep	328	143	43.6	[42]
		Lowlands	Sheep	182	90	49.45	
		Highlands	Goat	288	212	73.61	
		Lowlands	Goat	132	92	69.69	

Ethiopia	Bishoftu, Elfora abattoir	Highlands	Sheep	150	78	52	[43]
		Lowlands	Sheep	112	40	35.7	
		Highlands	Goat	138	84	60.9	
		Lowlands	Goat	132	67	47.2	

Ethiopia	Central Oromia	Highlands	Sheep	66	38	57.6	[26]
		Lowlands	Sheep	24	6	25.0	
		Highlands	Goat	160	88	55.0	
		Lowlands	Goat	150	5	4.7	

**Table 5:** Prevalence of *Cysticercus tenuicollis* in slaughtered sheep and goats according to period of the year (season) in the tropics.

Country	Area of study	Type of animals	Period of the year	Examined number	Number infected	Prevalence (%)	Source
Iran	Kermansher abattoir	Sheep	Spring	1413	96	6.70	[7]
		Goat	Spring	133	14	10.53	
		Sheep	Summer	1702	48	2.82	
		Goat	Summer	180	11	6.11	
		Sheep	Autumn	1208	102	8.44	
		Goat	Autumn	175	13	7.43	
		Sheep	Winter	1427	76	5.33	
		Goat	Winter	159	14	8.8	

Egypt	Awsan slaughterhouse	Sheep	Spring	129	23	11.98	[28]
		Goat	Spring	135	32	23.7	
		Sheep	Summer	180	21	11.6	
		Goat	Summer	182	26	14.3	
		Sheep	Autumn	88	19	21.6	
		Goat	Autumn	38	28	73.7	
		Sheep	Winter	209	26	12.4	
		Goat	Winter	129	31	24.0	

Israel	Galilee, North Israel	Lambs	Rainy season⁣^∗^	90	36	40	[61]
		Kids		NA	NA	20	
		Goat		NA	NA	24	

Tunisia	Northeast Tunisia	Sheep	Dry season	1198	64	5.3	[11]
		Goat		33	6	8.9	

Iraq	Karbala abattoir	Sheep	Rainy season	80	27	33.7	[62]
		Goat	Rainy season	80	32	40	
		Sheep	Rainy season	80	43	42.65	
		Goat	Rainy season	80	44	53.7	
		Sheep	Dry season	40	12	33.14	
		Goat	Dry season	40	14	39.44	

Iraq	Sulaymaniyah province	Sheep	Rainy season⁣^∗^	3325	836	25.1	[19]

Iran	Darab slaughterhouses	Both	Dry season⁣^∗^	1489	82	5.5	[29]
			Rainy season⁣^∗^	1186	75	6.3	

Iraq	Al-Shuala abattoir, northeast region, Baghdad city	Sheep	Dry season⁣^∗^	30	5	50	[56]
		Goat	Dry season⁣^∗^	30	11	36.7	
		Sheep	Rainy season Rainy season	30	4	32	
		Goat	Rainy season	30	12	40	

Abbreviation: NA, not available.

⁣^∗^Season determined based on the stated duration of sampling.

**Table 6:** Direct financial loss and proportional distribution of *Cysticercus tenuicollis* on visceral organs of infected sheep and goats in the tropics.

Country	Specie	Visceral organs affected according to abundance of cysts					Estimated monetary losses per year	Source
		Most affected, (%)	Second most affected, (%)	Third most affected, (%)	Fourth most affected, (%)	Fifth most affected, (%)		
Iraq	Ovine	Omentum (NA)	Abdominal cavity (NA)	Liver (NA)	Spleen (NA)	NA	NA	[38]
	Caprine	Omentum (NA)	Abdominal cavity (NA)	Liver (NA)	Spleen (NA)	NA	NA	

Ethiopia	Ovine	Omentum (58.2)	Mesentery (10.8)	Liver (10.9)	Peritoneum (8.2)	Lungs (2.4)	65,269.89 US dollars	[23]
	Caprine	Omentum (62.7)	Mesentery (12.2)	Liver (11.1)	Peritoneum (10.80)	Lungs (2.8)		

Ethiopia	Ovine	Liver (40.2)	Omentum (18.6)	Peritoneum (13.4)	Lungs (8.3)	Diaphragm (6.19)	NA	[41]
	Caprine	Liver (26.1)	Omentum (19.8)	Peritoneum (19.8)	Lungs (9.0)	Diaphragm (4.5)	NA	

Ethiopia	Ovine	Liver (7.81)	NA	NA	NA	NA	2,798.548 US dollars	[44]
	Caprine	Liver (15.8)	NA	NA	NA	NA	197.448 US dollars	

Ethiopia	Ovine	Omentum (34.52)	Mesentery (23.09)	Peritoneum (13.33)	Liver (16.43)	Lungs (10)	178,693.04 US dollars	[40]
	Caprine	Omentum (33.92)	Mesentery (20.98)	Peritoneum (7.45)	Liver (9.02)	Lungs (5.68)	178,693.04 US dollars	

Iran	Ovine	Omentum (4.07)	Liver (1.28)	Diaphragm (0.50)	NA	NA	NA	[7]
	Caprine	Omentum (4.33)	Liver (2.63)	Diaphragm (2.32)	NA	NA	NA	

Ethiopia	Ovine	Omentum (25.2)	Liver (19.5)	Mesentery (17.2)	Peritoneum (10.7)	NA	NA	[43]
	Caprine	Omentum (30.4)	Liver (27.1)	Mesentery (17.5)	Peritoneum (12.1)	NA	NA	

Israel	Ovine	Liver (50)	Lungs	NA	NA	NA	NA	[61]
	Caprine	Omentum (47.8)	Liver (7.4)	Abdominal cavity (11)	Peritoneum (4.8)	Mesentery (1.3)	NA	

Ethiopia	Ovine	Omentum (34.2)	Liver (7.8)	Mesentery (3.7)	Lung (0.8)	Uterus (0.2)	NA	[3]
	Caprine	Omentum (39.0)	Liver (14.7)	Mesentery (5.7)	Lung (3.0)	Uterus (0.0)	NA	

Egypt	Ovine	Omentum (62.92)	Mesentery (12.36)	Liver (11.24)	Urinary bladder (9)	Lung (2.25)	NA	[28]
	Caprine	Omentum (67.52)	Mesentery (21.37)	Liver (11.11)	NA	Lung (0.0)	NA	

Tanzania	Ovine	Lungs (46.7)	Liver and lungs (26.7)	Liver (20.0)	NA	NA	NA	[18]
	Caprine	Lungs (35.0)	Liver and lungs (25.0)	Liver (25.0)	Lung and Spleen (5.0)	NA	NA	

Algeria	Ovine	Liver (84.5)	Omentum (5.9)	Liver and omentum (9.6)	NA	NA	NA	[15]
	Caprine	Omentum (41.4)	Liver (41.0)	Liver and omentum (17.4)	NA	NA	NA	

Tunisia	Ovine	Mesentery (96)	Liver (2.0)	NA	NA	NA	NA	[11]
	Caprine	Mesentery (100)	NA	NA	NA	NA	NA	

Iraq	Ovine	Omentum (36.9)	Mesentery (34.1)	Liver (18.7)	Diaphragm (6.7)	Gall bladder (1.6)	NA	[31]

Ethiopia	Ovine	Omentum (31.1)	Liver (14.4)	Abdominal cavity (7.8)	Peritoneum (2.2)	Mesentery (0.0)	51,428.57 US dollars	[26]
	Caprine	Omentum (47.8)	Liver (7.4)	Abdominal cavity (11.3)	Peritoneum (4.8)	Mesentery (1.3)	51,428.57 US dollars	

Ethiopia	Ovine	Mesentery (19.4)	Peritoneum (19.8)	Liver (22.4)	Omentum (18.3)	Carcass (18.3)	34,885.9 US dollars	[5]
	Caprine	Mesentery (20.5)	Peritoneum (18.3)	Liver (18.1)	Omentum (17.2)	Carcass (21.6)	34,885.9 US dollars	

Bangladesh	Caprine	Peritoneum (7.9)	Liver (4.4)	Urinary Bladder (0.6)	NA	NA	NA	[59]

Abbreviation: NA, not available.

**Table 7:** Challenges for control of *Cysticercus tenuicollis* in sheep and goats in the tropics.

Geographical site	Challenge to control measures reported	Source
Basrah abattoir, Iraq	Uncontrolled defecation of stray dogs and wild carnivore population	[38]
Dire Dawa, Ethiopia	Large population of stray dogs, backyard/clandestine slaughtering and poor disposal of infected viscera, dogs not dewormed	[40]
Dessie municipal abattoir, Ethiopia	Presence of stray dogs and inadequate application of procedures of meat inspection	[42]
Bishoftu, Elfora Abattoir, Ethiopia	Inappropriate disposal of infected offal at abattoirs	[48]
Soroti municipal abattoir, Eastern Uganda	Poor husbandry management of livestock, poor resource communities, poor sanitary practices	[50]
Beit Dagan, Israel	Harvest of hay from the fields frequently shared with stray dogs and Jackals to feed lambs	[61]
Helimex abattoir, Ethiopia	Conducting strategic regular deworming, careful ante and post slaughter examination and burning or burying of condemned organs and carcass to avoid access to dogs and wild canids to break down the transmission cycle	[46]
Jeddah, Saudi Arabia	Dogs and domestic animals are the main reservoir for metacestodes in rural communities	[48]
Addis Ababa in Ethiopia	Variation in climate and poor husbandry practices	[3]
El Tarf abattoir, Algeria	Presence of stray dogs	[36]
Ngorongoro, Malambo slaughter slab Tazania	Presence of large dog population and other wild canids; practice of home/clandestine slaughter; slaughter slabs are not fenced and easily accessible to stray dogs; no dog helminth control strategy	[18]
Bishoftu Elora export abattoir, Oromia, Ethiopia	Presence of stray dogs; livestock including sheep and goat husbandry is widespread	[5]
Slaughterhouse, Northeast Tunisia	Higher infection; contaminated small scale, communal fields and farms, very limited veterinary health care and stray dogs	[11]
Oromia, Ethiopia	Inappropriate disposal of abattoir materials and easy access of stray dogs	[26]
Pakistan	Poor husbandry management; unorganized slaughtering and inappropriate waste disposal; higher prevalence of cysts	[63]
Sulaymaniyah province, Iraq	Poor resource communities, poor sanitary practices	[31]
India	Widespread illegal backyard/clandestine slaughtering of animals without veterinary supervision	[64]
Tamale metropolis abattoir, Northern Region Ghana	Poor cysts disposal, availability of stray dogs in slaughterhouse vicinity, predominance of free range system of production of goats and sheep	[57]
Al-Shuala abattoir, Northeast Region, Baghdad city	Dogs ingestion of the waste of infected animals; contamination of pastures	[56]

**Table 8:** Control strategies for the *Cysticercus tenuicollis* in sheep and goats in the tropics.

Area of focus	Control measure	Source
Control of stray dogs	• Preventing infected dogs (particularly stray dogs) from defecating on pastures grazed by livestock including sheep and goats is important in lowering the incidence of *C. tenuicollis* in sheep and goats whose final hosts are carnivores, mainly dogs • Reducing stray dog population would eliminate the contamination of pasture and livestock environments with eggs/segments of taenia hydatigena	[5, 7, 15, 23, 26, 36, 40, 41, 43, 49, 62]

Backyard slaughter	• Avoidance of backyard/clandestine slaughter to allow veterinary inspection and detection of the metacestodes in infected slaughter animals	[41, 43]

Proper disposal of contaminate materials	• Immediate attention to the safe and controlled elimination of all condemned abattoir materials • Avoid feeding owned and stray dogs contaminated offal's and organs of sheep and goats	[5, 7, 15, 23, 26, 31, 41, 43, 62]

Create awareness programs	• Awareness creation programs for the butchers, abattoirs workers, meat sellers, and dog owners about the danger of the metacestodes for human and animal health	[5, 7, 23, 26, 40, 43, 49]

Regular deworming of owned and stray dogs	• Strategic chemotherapeutic applications with appropriate broad spectrum anthelmintics	[5, 7, 23, 26, 41, 46, 49, 62]

Veterinary meat inspection	• Revision and upgrading of meat inspection legislation/procedures • Intensification of veterinary meat inspection and processing of meat products	[18, 40, 43]

One Health approach	• Awareness programs to collaborate in the One Health approach with involvement of all animal professionals in effectively resolving disease issues • Involvement of community participation, veterinary services, public health and environmental sectors to management health issues at the human–animal–environment interfaces	[18]

Research	• Knowledge gaps should be identified and prioritized for research • Updating information relating to epidemiology, diagnose and control of the disease • Resolve conflicting information • Provide data on cost benefit analysis of the disease	[23, 40, 65]

## Data Availability

The articles used in this review have been appropriately credited and cited for accessibility. The extracted data from chosen articles supporting the findings of this review are presented within the manuscript and are available from the corresponding author upon reasonable request.
